# Uncertainty Analysis of Biogas Generation and Gas Hydrate Accumulations in the Baiyun Sag, South China Sea

**DOI:** 10.3390/microorganisms13010005

**Published:** 2024-12-24

**Authors:** Pibo Su, Jinqiang Liang, Huai Cheng, Yaoyao Lv, Wei Zhang, Zuofei Zhu

**Affiliations:** 1 Sanya Institute of South China Sea Geology, Guangzhou Marine Geological Survey, China Geological Survey, Sanya 572025, China; spb_525@sina.com (P.S.);; 2 Academy of South China Sea Geological Science, China Geological Survey, Sanya 572025, China; 3 National Engineering Research Center for Gas Hydrate Exploration and Development, Guangzhou 511458, China

**Keywords:** natural gas hydrate, biogenic gas, South China Sea, gas hydrate distribution, resource estimation

## Abstract

In this study, we use petroleum systems modeling (PSM) to quantitatively simulate the uncertainty of biogenic gas generation modes and their impact on the spatial distribution and resource assessment of gas hydrates in the Baiyun Sag, South China Sea. The results are as follows: (1) Biogenic gas generation is significantly affected by thermal state and organic matter type. Low temperature is a primary reason for gas hydrate occurrence in shallower sediments when sufficient methane gas is present. This may be due to higher thermal conductivity of the overlying sediments, slower sediment burial rates, or other geological processes. (2) Natural gas hydrate resources are significantly controlled by biogenic gas generation. In addition to the thermal conditions of the source rock or sediment, the nature of the organic matter is another crucial factor. Generally, low-temperature methanogens produce more methane gas because they require less energy, whereas high-temperature methanogens require more energy and thus produce less methane gas. (3) The biogas generation thermal model is key to controlling the location and quantity of natural gas hydrate resources. The three possible gas-phase models, K0, K1, and K2 (representing different methanogens), produce varying amounts of methane gas over time, resulting in different amounts of natural gas hydrate resources. Additionally, the preservation of various methanogens in biogas source rocks can alter reservoir formation locations, influencing the scale and genetic model of natural gas hydrate resources.

## 1. Introduction

Natural gas hydrates (NGHs) are recognized as a high-efficiency and clean energy source with abundant resources. Significant achievements have been made in the exploration and trial production of gas hydrate worldwide, e.g., the Alaska North Slope, USA [1], Mackenzie Delta, Northwest Territories, Canada [2,3], eastern Nankai Trough, Japan [4,5,6,7,8,9,10], and northern South China Sea, China [11,12,13,14]. However, there are still some controversies on the gas source and amount [15,16,17], gas hydrate space distribution, and production strategy [18,19]. Numerous studies have shown that the high-concentrated gas hydrate ore body mainly comes from biogenic gas and thermogenic gas produced by the degradation of organic matter in sub-seabed sediments [20,21]. Biogenic gases, originating from bacterial activity, form early in a basin’s history at low temperatures, whereas thermogenic gases are typically generated later, at greater burial depths and higher temperatures. [22]. Due to their different origins, distinct exploration strategies are required for biogenic and thermogenic gases, as well as their associated resources, such as gas hydrates. In this paper, the authors aim to present the uncertainties of biogenic gas generation and its impact on the spatial distribution and resource estimation of gas hydrates in the Baiyun Sag, South China Sea.

It has been concluded that the gas source of most hydrates worldwide is biogenic gas; however, many unsolved problems remain in the process of biogas formation [14,15,16]. Coring in the Dongsha Sea area during the GMGS2 hydrate drilling voyages enables biogas production experiments in laboratories. These experiments study the production characteristics, laws, and controlling factors of biogas generation, providing an experimental basis for hydrate gas source analysis and biogas source rock evaluation [23]. According to the geochemistry data [15], a gas generation kinetic model is created [14]. This model revealed that the maximum generation of biogenic gas occurs at 34 °C. However, like any other method, the uncertainty of the biogas generation model is inevitable. This uncertainty may arise from errors in laboratory tests, the representativeness of lab conditions, or the selection of samples. Su, et al. [23] believe that variations in the characteristics and biomass of methanogens in the samples may be the main reason for differences in gas yield at different depths, even at the same temperature. Many researchers’ work has provided evidence for the variety of microorganisms in nature [24,25,26,27,28].

Geochemistry is widely used to study biogas source rocks and the gas they generate. Methanogenesis does not effectively begin in marine systems until pore-water sulfate has been significantly reduced and sulfate reducers are no longer active. The depth at which this transition occurs is controlled by the availability of sulfate, the burial rate, and the nature of the organic matter [22]. An equation for the rate of bacterial methanogenesis as a function of temperature makes the development of a qualitative cumulative methane generation calculation possible. Previous studies have typically used a constant thermal gradient and sedimentation rate to calculate the thermal regime [22]. However, the petroleum systems modeling (PSM) approach provides a better method for biogas generation modeling by integrating geological forward modeling algorithms. With this PSM approach, biogas generation is calculated using a reasonable sedimentation rate and basal thermal gradient based on basin analysis and modeling.

Various studies have demonstrated that the petroleum systems modeling (PSM) technique is a practical approach for testing the uncertainties of geological and geochemical issues [29,30]. This study employs PSM to simulate the effects of the biogas generation model and its associated controls on gas hydrate distribution and resources.

## 2. Regional Geological Background

**Figure 1:**
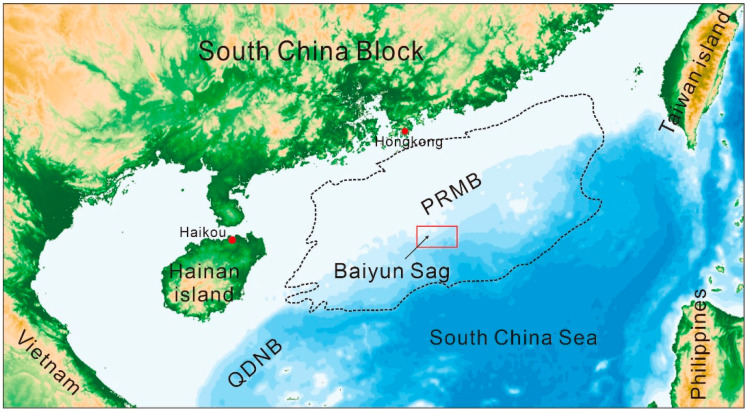
Study location in the PRMB (modified from Su, et al. [31] and Zhang, et al. [32]. PRMB—Pearl River Mouth Basin, QDNB—Qiongdongnan Basin.

**Figure 2:**
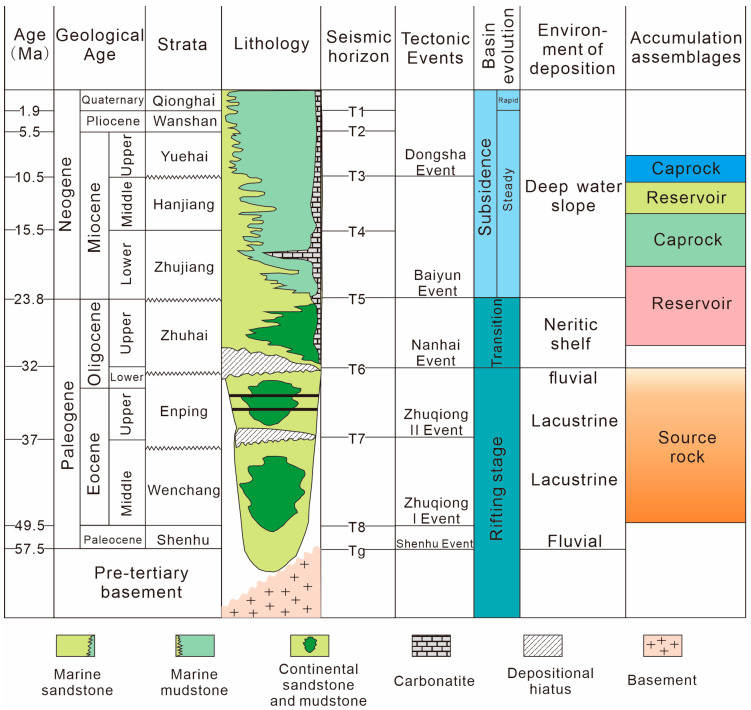
Major tectonic events, lithology, and sea level change in the Baiyun Sag, Pearl River Mouth Basin, modified from Zhang, et al. [33].

The study area is located within the Baiyun Sag, in the southern part of the Pearl River Mouth Basin (PRMB), with a water depth of 200~3000 m (Figure 1). The PRMB is a large petroliferous basin on the northern slope of the South China Sea. Tectonically, the Baiyun Sag experienced rifting and post-rifting stages and sedimentation transition from continental settings to marine settings in accordance with tectonic evolution. The rifting stage occurred approximately from the Late Cretaceous or Paleogene to the Early Oligocene. The post-rifting stage lasted roughly from the Late Oligocene to the present. Tectonic movement events, including Zhuqiong I, Zhuqiong II, and Baiyun, have occurred since the Paleogene. Over 10 km of sediments have been deposited in the center of the sag (Figure 2) [23].

The study area has been tectonically stable since 5.5 Ma. Some faults penetrate the gas hydrate stability zone. Generally, these faults exhibit larger normal offsets when the sedimentation rate is higher, indicating significant sedimentary supplies. During the tectonic evolution stages of the Paleocene and Early Oligocene, the Baiyun Sag received substantial deposits. The Shenhu Formation (Paleocene), Wenchang Formation (Eocene), and Enping Formation (Lower Oligocene) are interpreted as the three major source rocks in the basin. Since the Late Oligocene, the Baiyun Sag has entered the depression stage, the Zhuhai Formation, Zhujiang Formation, Hanjiang Formation, Yuehai Formation, Wanshan Formation, and Qionghai Formation in Quaternary were deposited and oil and gas were discovered in these intervals, and gas hydrates were drilled and observed in the shallower Qionghai Formation. The gas hydrates penetrated by the drilling are distributed in multiple layers, mainly in the range of 8~94 m below the sea floor (mbsf), and the thickness of a single layer is between 15 and ~35 m [31].

## 3. Data and Methods

### 3.1. Seismic Data and Structural Framework

**Figure 3:**
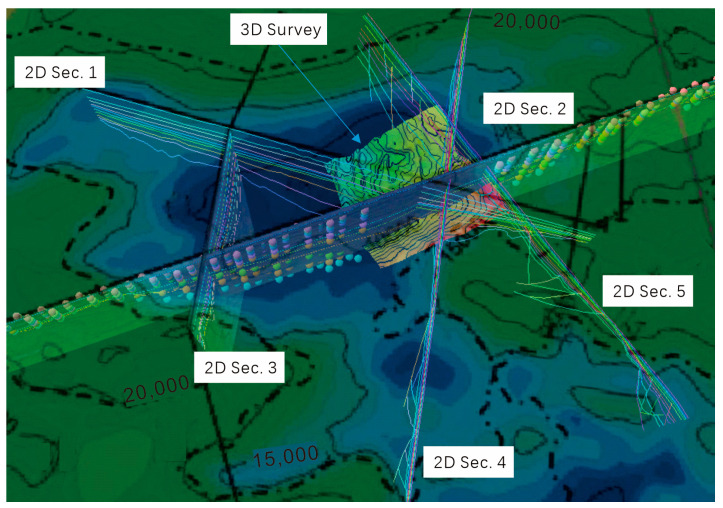
Database of the 3D structural model in this study.

All available data are utilized to establish the geological model framework (horizons in the depth domain), which includes three interpreted seismic sections (2D Sec. 1–3) from the literature, two sections of 2D seismic survey (2D Sec. 4–5) from the Guangzhou Marine Geological Survey (GMGS), and a 3D seismic survey from GMGS (Figure 3). Three sections (2D Sec. 1–3) from the literature are digitized, and the horizons are integrated with other data to construct the framework of the geological model. Due to the various data sources, the data quality varies. Discrepancies and mismatches between the data are resolved by following a priority or reliability order: 3D seismic survey, 2D seismic survey, and digitized section data from the literature. These various data sources cover the entire Baiyun Sag, which is located in the southern part of the Pearl River Mouth Basin (PRMB)

**Figure 4:**
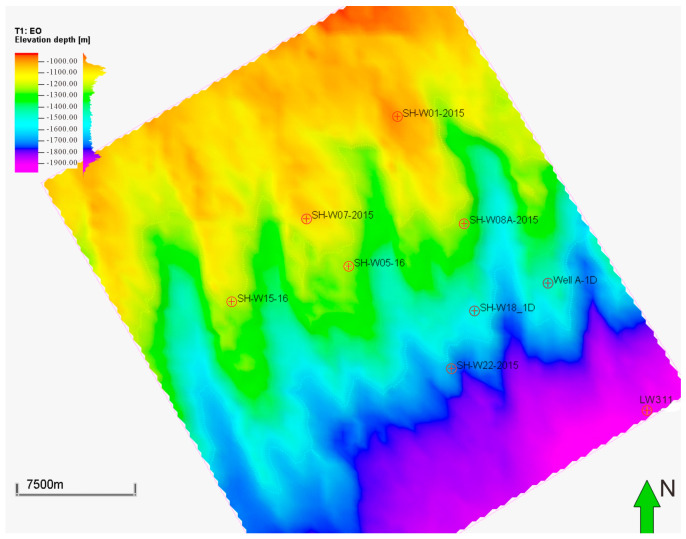
Structural map of targeted interval top: QH2 (portion of 3D seismic survey in the 3D PSM model).

Nine horizons from seabed (T_0_) to basement (T_g_) were interpreted based on the newly processed 3D seismic data (Figure 4). The age for each horizon is assigned according to the stratigraphic column (Figure 2). Lithology, free gas, and gas hydrate saturations are obtained from petrophysical interpretation for all available wells. The 3D properties are populated by integrating well data with 3D seismic attributes and inversion data.

### 3.2. Well Data and Key Parameters

Approximately 25 shallow wells were drilled to investigate gas hydrate formations within the area covered by 3D seismic data. Various petrophysical data, including resistivity image logs, caliper logs, gamma ray logs, neutron-density logs, sonic compressional and shear slowness logs, neutron capture spectroscopy, and nuclear magnetic resonance data, were integrated to comprehensively evaluate the gas hydrate reservoir [34]. Logging data can be used to identify and evaluate gas hydrate-bearing zones while segregating free gas zones based on parameters such as mineral composition, porosity, and gas hydrate saturation. Five types of lithology, including sandstone, siltstone, shale, limestone, and granite (basement), have been interpreted from logs and used in the 3D PSM model. The compaction curve for each lithology in PetroMod 2021 is used and calibrated with porosity and pressure data.

These petrophysical interpretation results can be used as input or calibration data for the PSM model. The modeled porosity, permeability, pore pressure, gas hydrate saturation, and gas saturation of associated gas accumulations must closely match the petrophysical interpretations to validate the reliability of the PSM model.

Source rock parameters, including total organic carbon (TOC), hydrogen index (HI), and hydrocarbon generation kinetics, are derived from organic geochemistry core sample testing [23,35]. Additionally, vitrinite reflectance (Ro) and temperature data from laboratory testing are typically used to calibrate the thermal history of the PSM model.

**Figure 5:**
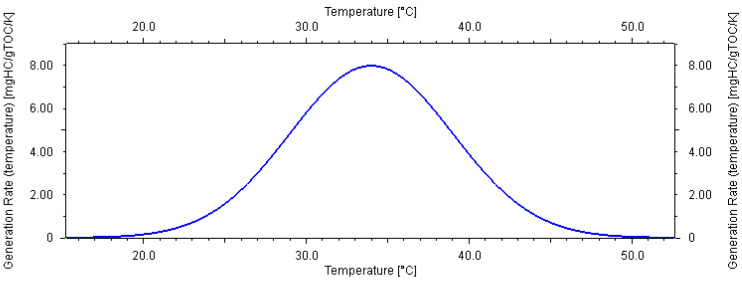
Medium temperature model of biogas generation (K0) used in this study.

Like all other parameters of a geological model, the biogas-generated hydrocarbon model inherently contains uncertainties because it is based on laboratory tests of rock samples. It is impossible to exactly replicate the subsurface conditions during geological history when hydrocarbons were generated. Most researchers believe that the kinetics model is typically temperature-dependent [4,5,14,22,23,36,37]. Based on the geochemistry laboratory tests of local samples, the main peak temperature of methane generation in this area is 34 °C (Figure 5). An analysis of the pyrolysis experiment results [23] concludes that three key factors affect the formation of biomethane in sediments: the richness of organic matter, the activity of methanogens, and environmental factors. During the biochemical gas production stage, the methane production rate is closely related to temperature. In the study area, the highest methane production occurred between 20 °C and 40 °C, and production ceased at temperatures above 55 °C.

**Figure 6:**
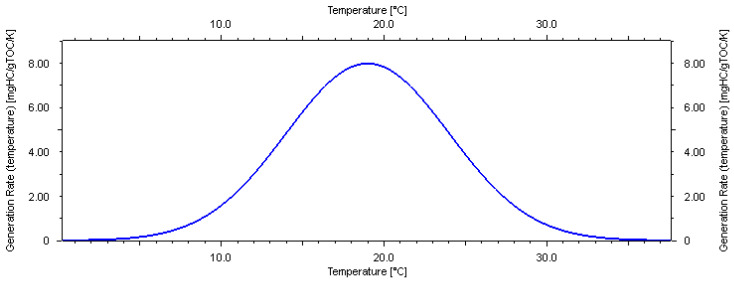
Low-temperature model of biogas generation (K1) used in this study.

**Figure 7:**
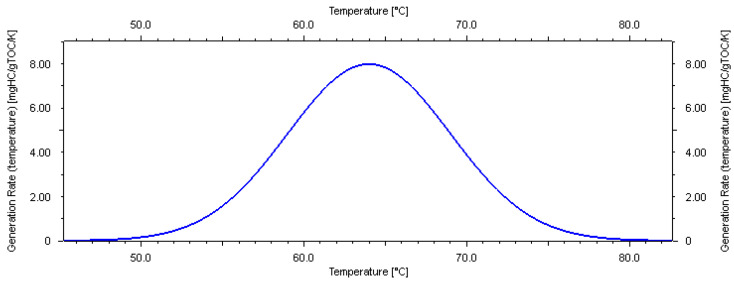
High-temperature model of biogas generation (K2) used in this study.

Fujii, et al. [4] used a model with a peak temperature of 12.5 °C based on laboratory-derived pentamethylicosane (one of the biomarkers for methanogenic archaea) analytical results from core samples. Considering these literature research results and the uncertainty of laboratory testing, three possible kinetics models are proposed in this study (Figure 5, Figure 6 and Figure 7).

For the shallow biogas-generated hydrocarbon model, which is the main gas source of hydrate in this area, the analysis and laboratory data indicate that the main peak temperature of generation is 34 °C (medium temperature type K0, the master model). Based on these findings, combined with research results from the Chinese and English literature, two other possible bioaerodynamic models are proposed in this study: the low-temperature K1 model and the high-temperature K2 model. The low-temperature model (K1) suggests that biogenic source rocks require less energy to generate biogenic gas, with peak generation occurring at around 18 °C. In contrast, the high-temperature model (K2) indicates that biogenic source rocks require more energy to generate biogenic gas, with peak generation occurring at around 64 °C.

### 3.3. PSM Model Building and Simulations

**Figure 8:**
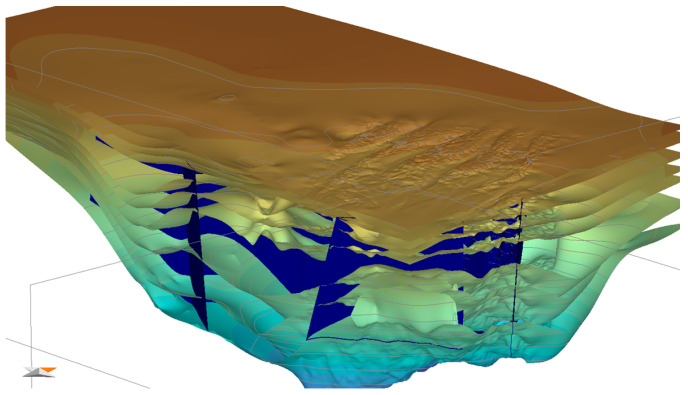
Framework of the 3D PSM model. Faults are displayed in blue.

The 3D geological model properties, such as lithology and source rock parameters, including total organic carbon (TOC), hydrogen index (HI), and source rock kinetics, are from the available seismic and drilling data (Figure 8). Additionally, geological ages were assigned to each formation. Finally, boundary conditions, including heat flow (HF), paleo water depth (PWD), and sedimentary water interface temperature (SWIT), were established based on previous study results from public papers [15,29,38].

**Figure 9:**
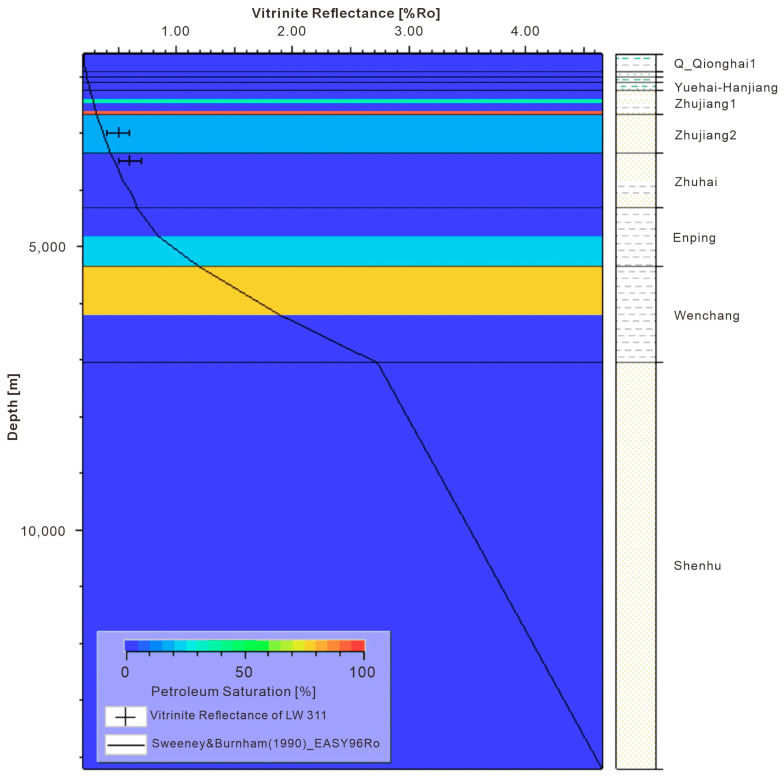
Ro calibration at well L311. The background is the overlay of petroleum saturation.

**Figure 10:**
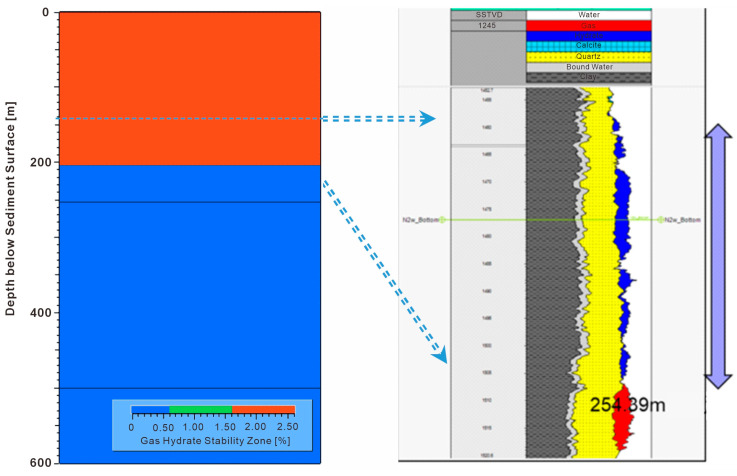
Gas hydrate stability zone (GHSZ) simulation results compared with petrophysical interpretation results in the W17 well.

After structural model building, property population, and boundary condition setting, the first step is to run thermal simulations. Once the simulation results are calibrated with known Ro and temperature data from wells (Figure 9), the porosity and pore pressure of model results are calibrated with available well logging data and well testing. Figure 9 shows that both the shallower (Zhujiang Formation) and deeper (Enping and Wenchang Formation) formations have hydrocarbon accumulations. Secondly, gas hydrate stability zone (GHSZ) simulation and calibration with well data are conducted (Figure 10). The 3D GHSZ distribution, its associated free gas accumulations, and all other conventional hydrocarbon accumulations are obtained from the GHSZ simulation. The 3D modeling results can be compared with petrophysical interpretations. As we can see, the simulated GHSZ is thicker than the gas hydrate thickness interpreted from petrophysical data. This makes sense because gas hydrates only form when sufficient gas is available.

A scenario analysis was performed after the master model results were calibrated, as described above. Two scenarios were simulated using different biogas generation models: low-temperature K1 and high-temperature K2.

## 4. Results and Discussion

Numerous studies have shown that the gas in natural gas hydrate deposits primarily originates from biogenic and thermogenic gas generated from the organic matter in sediments. However, due to their different origins, the extent of each type’s contribution to hydrate formation remains unclear. This uncertainty directly impacts the evaluation of hydrate resource potential and the planning of subsequent exploration. This study numerically demonstrates the effects of various biogas generation models on the amount, timing, and locations of gas hydrate formation. It serves as the first warning to gas hydrate researchers that significant uncertainties exist and is potentially the largest factor affecting gas hydrate resource estimations and explorations.

**Figure 11:**
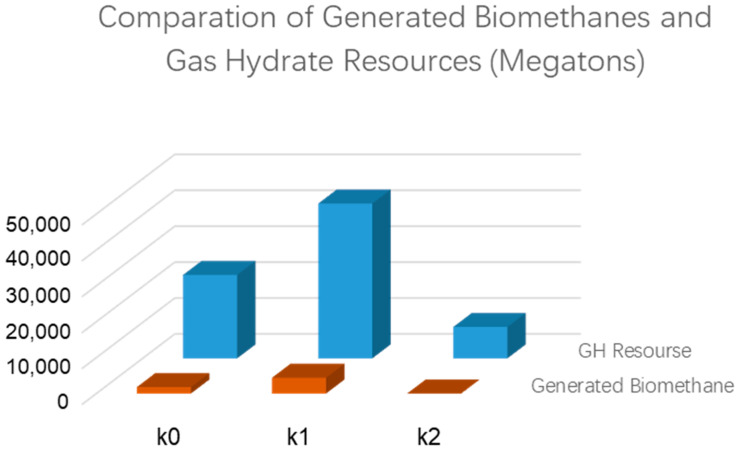
Comparison of generated biomethanes and associated gas hydrate resources using various models.

In addition to the biogas generation model, the amount of biogas generated changes depending on many other geological and geochemical factors, as well, such as the total organic matter, underground temperature, formation water salinity etc. For numerical simulation studies, the generation model is simplified to capture the main parameters of the entire process. The amount of gas derived from a model is likely to be uncertain. This paper proposes various models of the key factors to analyze the associated uncertainties. The peak temperature of the biogas generation is critical to evaluate. The total gas generation related to hydrate resources in the region ranges from K2 to K1 (Figure 6 and Figure 7), with K0 as the primary model representing the most likely intermediate scenario. K1 and K2 are considered near-extreme cases. (Figure 11).

**Figure 12:**
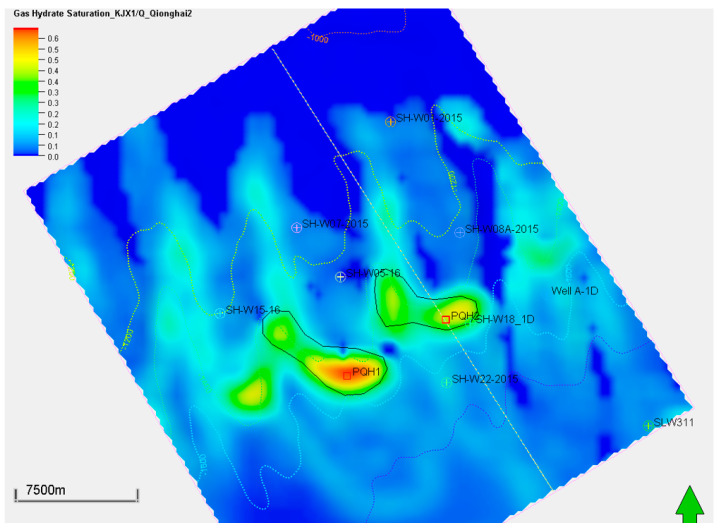
Gas hydrate saturation distribution of the targeted interval of QH2 using the low-temperature generation model (K1). The green arrow represents the North.

**Figure 13:**
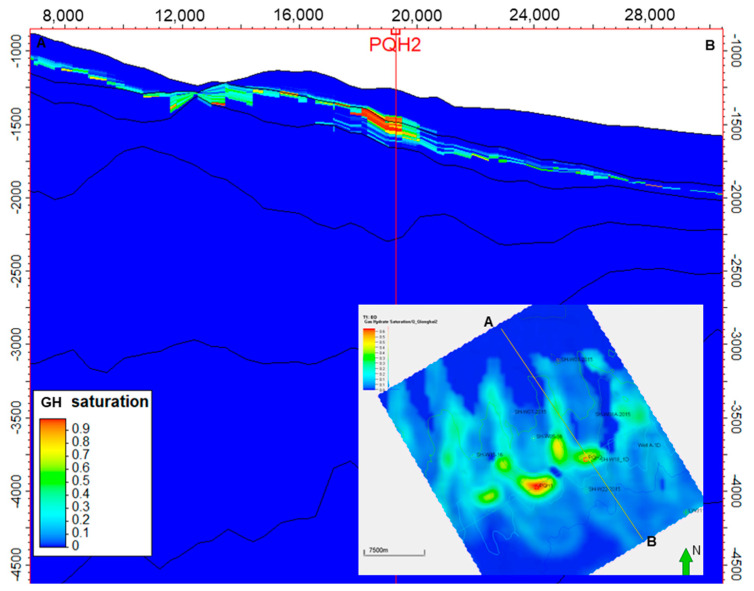
Gas hydrate saturation distribution (section AB) derived from the low-temperature model (K1). The green arrow represents the North.

Although the low-temperature model (K1) generated the most significant biogenic methane and associated methane hydrate, the model predicted a similar sweet spot in the 3D space. Both the K0 and K1 models predicted the PQH1 and PQH2 prospects displayed in Figure 12 and Figure 13. The figures indicate the hydrate sweet spots are approximately aligned with the reservoir top structure maps. However, the formation of gas hydrate is controlled by many factors, such as the surrounding temperature, pressure, and gas availability. The reservoir top structure is just one of the factors influencing hydrate formation.

**Figure 14:**
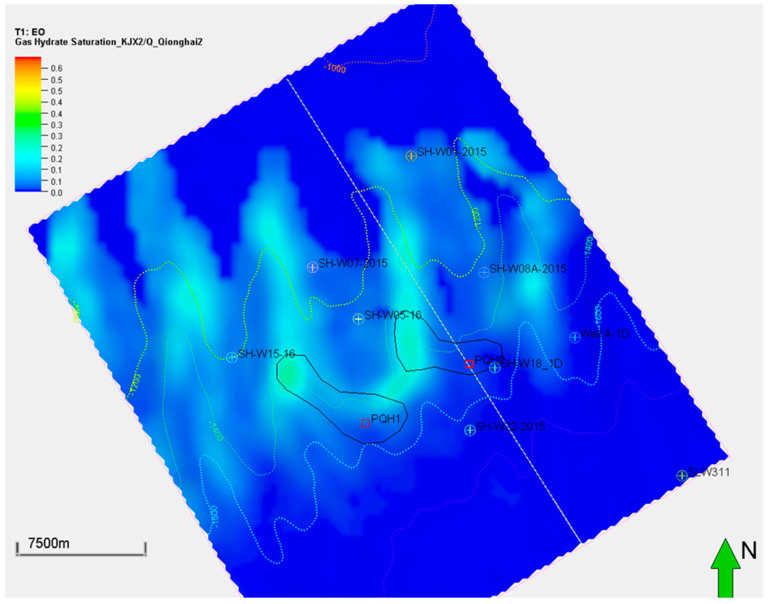
Gas hydrate saturation distribution of the targeted interval of QH2 using hte high-temperature generation model (K2). The green arrow represents the North.

**Figure 15:**
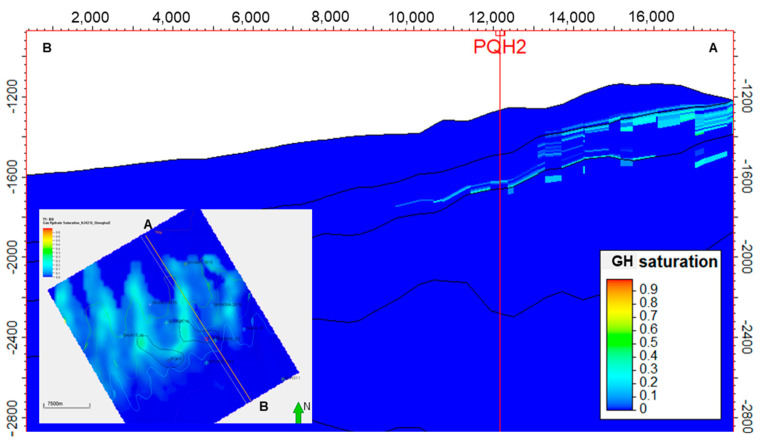
Gas hydrate saturation distribution section derived from the high-temperature model (K2).

Compared to the low-temperature model, the high-temperature model (K2) did not predict similar prospects to the K0 and K1 models because it requires more energy to generate biogenic methane and the associated hydrate (Figure 14 and Figure 15). The PQH1 and PQH2 prospects did not show sufficient hydrate formation in the K2 model results. It is clear that despite having the same geological conditions, hydrate sweet spots can change if the biogas generation model is not accurate enough. The biogas generation model is usually derived from laboratory tests based on samples from drilling, seafloor sediments, and/or lab cultivation. Uncertainty is inevitable during laboratory tests for biogas generation.

**Figure 16:**
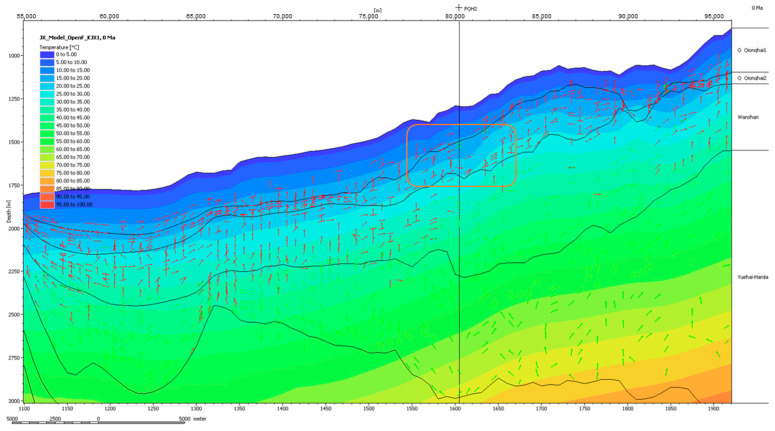
Temperature distribution (section AB in the Figure 13) results derived from the low-temperature model simulation (K1).

Gas hydrates occur in places where the surrounding temperature is low and the pressure is high. A close examination of the PQH2 prospect, predicted by both the low and intermediate models (Figure 16), shows that PQH2 has a relatively lower temperature than the surrounding environment. This lower temperature can be attributed to many geological factors. One possible reason is the high thermal conductivity of the overlaying sedimentary layers or the reservoir layer itself. In this case, the thermal isocline of the prospect area bends downward, and the temperature is lower than the surrounding condition. This is one of the main reasons that methane hydrate accumulates in the PQH2 prospect.

**Figure 17:**
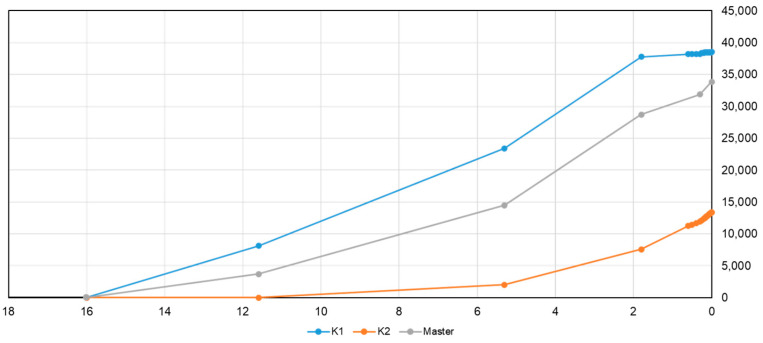
Biogenic gas generation history of Hanjian-Yuehai.

The timing of biogenic gas generation varies depending on the biogenic generation model used (Figure 17). The K1 and Master models initiate biogenic gas generation earlier, with peak generation times ranging from approximately 6 to 2 million years ago (Ma). In contrast, the K2 model’s peak biogenic gas generation begins after 2 Ma.

The formation of gas hydrates is influenced by a combination of formation pressure, temperature, and the availability of biogenic gas. Therefore, the precise timing of biogenic gas generation and the geological history of the reservoir layers (including porosity, permeability, pressure, and temperature) must align perfectly to enable the occurrence of gas hydrates.

Additionally, another perspective on gas hydrate formation is worth discussing. The possible range of compositions of biogas specimens could include methane, carbon dioxide, propane, or other gases. These ranges can fluctuate based on the type of organic material used, the specific anaerobic digestion process, and other environmental factors. A more significant point is that when components other than methane are involved in gas hydrate formation, the thermodynamic and kinetic models of gas hydrate will change, as well [39,40,41]. Some researchers have found that the addition of propane significantly lowers the pressure required for hydrate formation compared to pure methane hydrates. This could be attributed to the capability of different species to occupy the small or large cages of hydrate cubic structures [42]. In natural gas hydrate formation, it is likely that more than one component, such as methane, is involved. Therefore, another uncertainty associated with the gas hydrate formation model cannot be ignored. This could be a future project or paper.

## 5. Conclusions, Implications, and Future Works

The PSM modeling results show that biogenic gas generation is significantly affected by the thermal regime and organic types. It is well known that gas hydrates form when gas is trapped under conditions of low temperature and high pressure. The simulation results indicate that in shallower sediments, in which rocks are usually not fully compacted, the formation pressure is typically hydraulic. Low temperatures become one of the main reasons that gas hydrates form when sufficient methane gas is available. The low temperature could be caused by the high thermal conductivity of the overlying sediments, the slow burial rate of sediments, or other geological processes

Gas hydrate resources are significantly controlled by the amount of biogas generated. In addition to the thermal condition of the biogas source rocks or sediments, the nature of the organic matter is another important factor controlling biogas generation. Certain organic matters generate biogenic methane at low temperatures, while others generate biogenic methane at elevated temperatures. Generally, low-temperature methanogens produce more biogas since they require less energy to generate methane, whereas high-temperature methanogens produce less biogas since they require more energy to generate methane.

The biogas generation thermal model is a critical factor in controlling gas hydrate accumulation locations and resource amounts. Other geological factors, such as thermal history, lithology distributions, and pressure regimes, have similar effects. The simulations in this study show that the three possible biogas generation models (K0, K1, and K2) produced different amounts of biogas at various times, ultimately generating different amounts of gas hydrate resources. Additionally, the accumulation locations can also change depending on the types of methanogens preserved in the biogas source rocks.

One significant implication of this study is that gas hydrate resource estimations should be approached with caution, as many factors influence them. The biogas generation model is one such factor. PSM is a practical tool for testing uncertainties based on a reliable geological model. The author suggests that resource estimation could be improved by integrating PSM modeling with gas hydrate production tests. Additionally, a cross-domain workflow should be performed and iterated until a calibrated model becomes available.

The multidisciplinary workflow can be initiated with geological and geophysical studies, ultimately delivering a reliable geological model. Geochemical laboratory tests are another pillar for improving the understanding of gas hydrate distribution and resource estimation. When both the geological model and an uncertainty analysis of biogas generation are available, PSM modeling can serve as the third pillar of the workflow, integrated with insights from other domains and production testing. The iterative workflow, incorporating multiple domains of understanding, ultimately provides a stable foundation for gas hydrate exploration and exploitation.

## Data Availability

The original contributions presented in this study are included in the article. Further inquiries can be directed to the corresponding author.
